# Residence-colonization trade-off and niche differentiation enable coexistence of Escherichia coli phylogroups in healthy humans

**DOI:** 10.1093/ismejo/wraf089

**Published:** 2025-04-30

**Authors:** Thibaut Morel-Journel, Sonja Lehtinen, Olivier Cotto, Rafika Amia, Sara Dion, Clarisse Figueroa, Jonathan N V Martinson, Pascal Ralaimazava, Olivier Clermont, Xavier Duval, Forough L Nowrouzian, Seth T Walk, Erick Denamur, François Blanquart

**Affiliations:** Université Sorbonne Paris Nord, Université Paris Cité, INSERM, IAME, 9300, Bobigny, France; Department of Computational Biology, University of Lausanne, CH-1015, Lausanne, Switzerland; Plant Health Institute of Montpellier, INRAE, Univ Montpellier, CIRAD, Institut Agro, IRD, 34000, Montpellier, France; Université Paris Cité, Université Sorbonne Paris Nord, INSERM, IAME, 75018, Paris, France; Université Paris Cité, Université Sorbonne Paris Nord, INSERM, IAME, 75018, Paris, France; Université Paris Cité, Université Sorbonne Paris Nord, INSERM, IAME, 75018, Paris, France; Innovative Genomics Institute, University of California, 94720, Berkeley, CA, United States; AP-HP, Hôpital Bichat, Centre d'Investigation Clinique, INSERM CIC 1425, Université Paris Cité, 75018, Paris, France; Université Paris Cité, Université Sorbonne Paris Nord, INSERM, IAME, 75018, Paris, France; Université Paris Cité, Université Sorbonne Paris Nord, INSERM, IAME, 75018, Paris, France; AP-HP, Hôpital Bichat, Centre d'Investigation Clinique, INSERM CIC 1425, Université Paris Cité, 75018, Paris, France; Department of Infectious Diseases, Institute of Biomedicine, University of Gothenburg, Guldhedsgatan 10, Gothenburg S-413 46, Sweden; Department of Microbiology and Immunology, Montana State University, 59717, Bozeman, MT, United States; Université Paris Cité, Université Sorbonne Paris Nord, INSERM, IAME, 75018, Paris, France; AP-HP, Laboratoire de Génétique Moléculaire, Hôpital Bichat, 75018, Paris, France; CIRB, Collège de France, Université PSL, CNRS, INSERM, 75005, Paris, France

**Keywords:** coexistence, *Escherichia coli*life-history trade-off, Markov model, microbiota, survival analysis

## Abstract

Despite abundant literature on pathogenicity and virulence of the opportunistic pathogen *Escherichia coli*, much less is known about its ecological and evolutionary dynamics as a commensal. Based on two detailed longitudinal datasets on the gut microbiota of healthy adults followed for months to years in France and the USA, we identified a robust trade-off between the ability to establish in a new host (colonization) and to remain in the host (residence). Major *E. coli* lineages (phylogroups or subgroups) exhibited similar fitness but diverse strategies, from strong colonisers residing few days in the gut to poor colonisers residing for years. Strains with the largest number of extra-intestinal virulence associated genes and highest pathogenicity also resided for longest in hosts. Furthermore, the residence of a strain was more strongly reduced when it competed with other strains from the same phylogroup than from another phylogroup, suggesting niche differentiation between phylogroups. Based on a discrete-state Markov model developed to describe *E. coli* dynamics in a host population, we found that the trade-off and niche differentiation acted together as equalizing and stabilizing mechanisms allowing phylogroups to coexist over long periods of time. Our model also predicted that external disturbances may disproportionately affect resident strains, such as the extraintestinal pathogenic ones of subgroup B2.3. Our results call for further studies outside high-income countries, where the prevalence of this phylogroup is much lower. More generally, the trade-off between colonization and persistence could play a role in the diversification of other bacterial species of the microbiome.

## Introduction

Understanding how bacteria diversify under the combined action of mutation and recombination is a long-standing issue in microbiology and evolutionary biology [1, 2]. Multiple mechanisms have been proposed to explain the observed diversity and the coexistence of bacterial strains that may exhibit fitness differences. Trade-offs between life-history traits can enable species to diversify into distinct strategies [3, 4]. Differentiation between ecological niches among bacteria can also favour diversity by allowing distinct strains to exploit the heterogeneity between hosts [5, 6]. For instance, the combination of a serotype-specific and non-specific host immune responses can generate host differences allowing coexistence among *Streptococcus pneumoniae* serotypes [7]. Other mechanisms not involving selection, such as a recombination rate negatively correlated with genetic distance, can lead to genetically distinct groups as well [1, 8].

The question of coexistence is especially critical for species such as *E. coli*, which is not only a commensal bacteria of the human gut [9], but also an opportunistic pathogen. *E. coli* is responsible for various forms of diarrhoea [10, 11] and extraintestinal infections, including urinary tract [12, 13] and bloodstream infections (BSI) [14, 15], causing around one million deaths worldwide each year [16–18]. The species is highly diverse and displays a self-similar genetic structure at several phylogenetic levels [19, 20], including that of the “phylogroups” [21, 22], which are largely monophyletic lineages [9, 20, 23] that differ in their virulence and antibiotic resistance profiles [24, 25]. Commensal and extraintestinal pathogenic *E. coli* are not strictly phylogenetically separated [26] and the transition to infection is the result of complex and hardly predictable interactions between several genetic, host, and environmental factors [27]. Infections remain very rare events among the species, so that genes associated with pathogenicity (the probability to cause an infection) and virulence (the severity of infection) may evolve mainly as a result of their role in commensalism [28, 29]. Characterizing the dynamics of commensal *E. coli*, as well as their association with pathogenicity and virulence, is essential for understanding how the phylogenetic structure of *E. coli* emerged and for predicting the future evolution of the species.

Understanding *E. coli* diversity requires precise characterization of the life-history traits of *E. coli*, which define the epidemiological dynamics in the human population—the rates at which strains colonize new hosts are cleared from them. These dynamics can be quantified through longitudinal follow-up of *E. coli* in healthy subjects, which unfortunately remains rare. Studies rather focus on diseased individuals [30, 31], e.g. to assess the impact of antibiotic use on the microbiota [32, 33]. The initial colonization of infants during their first months of life has been studied [34–37], but longitudinal studies on healthy adults include a limited number of subjects [38–42].

The scarcity of longitudinal studies on healthy individuals can be explained by the complexity of their implementation. In addition to the need for a large number of subjects and of samples by subject, a single host can harbour several strains at the same time [43], multiplying the amount of work required to characterize each sample. In this study, we resolved this problem by using two complementary and very detailed longitudinal datasets to characterize the dynamics of *E. coli* in healthy adults of high-income countries. The first one, analysed in a previous study [44], includes eight subjects followed over months to years in the USA with an extremely thorough characterization of the different clones present at the same time in each host. The second dataset, original to this study, includes 46 subjects followed over four to five months in France. Based on these datasets, we aimed to (i) quantitatively characterize the life-history traits of *E. coli* phylogroups, (ii) correlate these commensal traits with those associated with pathogenicity and virulence in the species, and (iii) derive the consequences of our results for the epidemiological dynamics and potential coexistence of these phylogroups.

## Materials and methods

### Longitudinal datasets

We used two longitudinal datasets collected from adults living in high-income countries to perform the survival analyses. The first one, referred to as the French dataset, was original to this study. See the Supplementary material for further details about the data collection and a graphical representation of the longitudinal data (Fig. S11). This dataset included 415 clones collected from 46 healthy subjects in the Paris area over the course of 10 samples approximately every 2 weeks (14.9 days on average between samples, SD = 4.9 days), over periods of 111 to 135 days in 2021 and 2022. The clones were distinguished using multilocus variable number tandem repeat analysis (MLVA) [45]. Each clone was assigned to a phylogroup or a subgroup, based on the Clermont quadruplex PCR [22], followed by allele-specific PCR to identify phylogroups C [22], E [19, 22], and G [46] (Table 1). We considered phylogroups A (separated into subgroups A.1 and A.2), B1, B2 (separated into subgroups B2.1, B2.2, and B2.3), C, D (separated into subgroups D.1 and D.2), E, F, and G. Subgroups of phylogroups E and G were not considered, because of their rarity in the dataset. We considered these subgroups, which are phylogenetically consistent groups (Fig. S8) encompassing specific sets of sequence type complexes (STc), in order to stay consistent with the classification used in the USA dataset (see below), to increase the discriminatory power of the subsequent data analyses performed, and because of their epidemiological relevance. For example, the main STc responsible for BSI in phylogroups A (STc10) and D (STc69) [47] are respectively the main representatives of subgroups A.2 and D.1, and the STc in subgroup B2.1 (STc12 and STc28) are specifically uropathogenic [27].

**Table 1:** Delineation of the subgroups of phylogroups based on the Clermont quadruplex profile, representing the presence (+) or absence (−) of *arpA*, *chuA*, *yiaA*, and *TspE4.C2*, with the main sequence type complexes (STc) included in each of them, based on data from human commensal and pathogenic strains. Subgroups within phylogroup E and G were not considered separately, given the low number of clones from these phylogroups in the datasets considered.

Phylogenetic group	Clermont profile	Subgroup	Main STc
A	+ − – –	A.1	STc46, STc93, STc399
A	+ − + −	A.2	STc10
B1	+ − – +	BA	STc58, STc29
B2	– + + −	B2.1	STc12, STc28
B2	– + − +	B2.2	STc569
B2	– + + +	B2.3	STc131, STc73, STc95, STc127
C	+ − + −	C	STc88
D	+ + − –	D.1	STc69
D	+ + − +	D.2	STc38, STc405
E	+ + − –	E.1	STc11, STc543
E	+ + − +	E.2	STc57
E	+ + + −	E.3	STc219
F	– + − –	F	STc59, STc62, STc648
G	– + − –	G.1	STc117
G	– + − +	G.2	STc174

The second dataset, referred to as the USA dataset, was analysed in a previous study [44]. See the Supplementary material for a graphical representation of the longitudinal data (Fig. S10). This dataset included 117 clones collected from eight healthy subjects over periods ranging from 245 to 849 days (11.7 days on average between two samples, SD = 11.1 days). The clones were distinguished using GTG5 repetitive element amplification [48]. Clone were assigned to phylogroups or subgroups following the same method as for the French dataset and corresponding to the groups used in the original study [44]. We considered phylogroup A (separated into subgroups A.1 and A.2), B1, B2 (separated into subgroups B2.1, B2.2, and B2.3), C, D, E, and F. Phylogroups C and G did not appear in this dataset, respectively because a single clone of phylogroup C was recorded and was discarded, and because phylogroup G was not defined separately in the original study.

To assess whether our conclusions hold for colonization dynamics in infants, we considered a third dataset analysed in a previous study [36] and referred to as the infant dataset. See the Supplementary material for details about this dataset and the analyses performed and a graphical representation of the longitudinal data (Fig. S12). This dataset included 273 clones collected from 130 infants in Sweden at fixed times since birth (after 3 days, 1, 2, and 4 weeks, 2, 6, and 12 months). The clones were assigned to one of the four following phylogroups using the triplex Clermont PCR [21]: A, B1, B2, and D. For simplicity, all the phylogroups and subgroups considered in the different datasets are all referred to as ``phylogroups” in the following.

### Survival analyses

**Figure 1:**
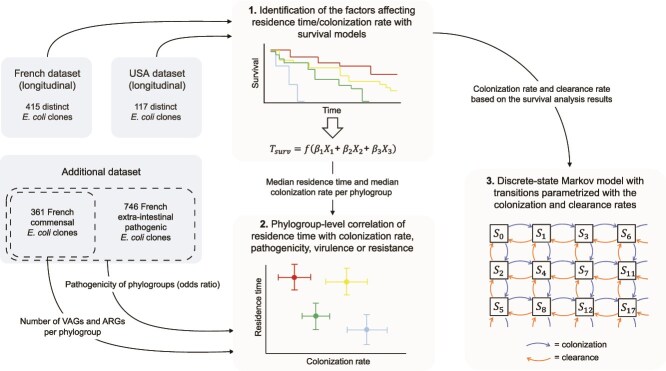
Schematic representation of the analyses performed in this study, in three steps: (1) survival analyses based on the longitudinal datasets to identify the factors affecting the residence time and colonization rate of the clones, (2) correlations at the phylogroup level of the residence time with colonization rate (both estimated from the survival analyses), pathogenicity (estimated from the additional dataset), the number of VAGs, and the number of ARGs (estimated from the commensals of the additional dataset), and (3) the discrete-state Markov model describing epidemiological dynamics of *E. coli* based on colonization and clearance rates parametrized with the survival analyses.

We used the software *R* [49] and the package *survival* [50] to perform survival analyses on the French and USA datasets (Fig. 1). We used parametric accelerated failure time (AFT) models, which described the relationship between the time to an event—colonization or clearance—and a set of explanatory factors [51]. This type of model takes into account interval-censored data, i.e. the fact that colonization or clearance can occur between two samples. We counted the residence time of a clone (time to clearance) starting from its first appearance in the host. We counted the time to colonization from the first appearance of the last preceding colonizing clone. For clarity, the results are presented in terms of the colonization rate, defined as the inverse of this time to colonization. We performed one analysis per dataset (French or USA) and per response variable (colonization rate or residence time). We excluded left-censored data, but included right-censored data (clones still present in the last sample of the host) for the AFT models of residence time.

Even though we assumed that residence time was impacted by within-host dynamics alone, we considered colonization to be the result of inter-host transmission. Therefore, the colonization rate of a phylogroup is affected by its frequency in the host population as a whole. To account for this, we corrected the times to colonization by the frequencies of phylogroups in their respective countries, based on external data (see Supplementary material). We then performed the survival analyses using these corrected data.

We considered how colonization and clearance depended on two qualitative factors—the host and the phylogroup of the focal clone—as well as the following quantitative factors: the total cell density of *E. coli* (in CFU/g), the number of clones inhabiting the host (other than the focal clone), and the number of other clones of the same phylogroup (other than the focal clone). We also considered the cell density of the clone considered for the AFT models of residence time in the USA dataset only. This information was not available for the French dataset owing to the smaller number of colonies typed per time-point. Because of the wide variations in cell densities observed between samples, we used their log_10_-transformed values.

We performed model selection based on Akaike’s information criterion (AIC, [52]) to identify the AFT model best explaining the residence times and times to colonization in the French and USA datasets. See the Supplementary material for details about the model selection. In each case, we considered the models including any combinations of the explanatory factors presented above, with the time to the event following one of the three following distributions: exponential, Weibull, and log-logistic. We ranked these models based on their ΔAIC, i.e. the difference between their own AIC and the smallest one, and kept all models with ΔAIC <2. We selected the most parsimonious model from this subset, unless a likelihood ratio test indicated that a more complex one from this subset explained the data significantly better. When discriminating between models with different distributions for the time to the event, we considered the exponential distribution to be the most parsimonious, as it assumed a constant hazard over time.

For both the French and USA datasets, we computed the estimate and standard error of median residence time and colonization rate of each phylogroup according to the AFT models. We defined the median colonization rate as the inverse of the estimated median time to colonization. To obtain estimates of typical colonization rates and residence times for each phylogroup, we computed these estimates for an average host based on those included in the dataset and for the average values of all the quantitative explanatory factors included in the model. These estimates were used to compute the Spearman’s correlation coefficient between colonization rate and residence time. We used the associated standard errors of parameter estimates to generate confidence intervals by parametric bootstrapping. We randomly sampled these distributions to generate one million sets, including each one residence time and one colonization rate per phylogroup. We computed the Spearman’s correlation coefficient for each set, thus generating a distribution of the correlation between residence and colonization. We considered a correlation to be significant if 95% of the distribution of Spearman’s coefficients across bootstraps was different from zero.

### Association of residence time with pathogenicity, and the number of virulence-associated genes

We used an additional dataset, analysed in a previous study [53], to estimate the number of virulence-associated genes (VAG) and of antibiotic resistance genes (ARG) of each phylogroup, as well as their pathogenicity (Fig. 1). It included 361 commensal and 746 pathogenic clones of *E. coli* (causing BSI), collected between 2000 and 2017 in Paris and the North of France. These clones were assigned to the same phylogroups or subgroups as the ones described in the French dataset. These data also included the pathogenic status of each clone (1 for pathogenic, 0 for commensal), as well as the number of ARGs (out of a list of 40) and VAGs (out of a list of 368) carried by the commensal clones. Among VAGs, some were specifically associated with one of the following type of virulence factors classically associated with pathogenic *E. coli* [54–56]: toxins (149 genes), adhesin (115 genes), iron acquisition systems (50 genes), and protectins (10 genes). Information on the carriage of resistance and virulence genes was readily available, following the analysis of genomic sequences of the commensals described in the original study [53]. The ``toxin” category included genes involved in bacterial warfare, as well as in targeting human cells. To also assess the carriage of genes linked to interference competition, we created a sub-category of VAGs linked to toxins annotated as microcins, colicins, or as type IV or VI secretion systems, which are involved in bacterial warfare [57] (16 genes). We estimated the pathogenicity of each phylogroup based on this additional dataset, using a binomial regression with the pathogenic status as a response variable and the phylogroup as the explanatory factor. We used the odds ratio for the association between infection and phylogroup, as estimated from this statistical model, as a proxy for pathogenicity. We also estimated the number of genes (ARGs, total VAGs, and VAGs of each type) carried by the strains of each phylogroup on average, using a binomial regression. In each case, we considered the proportion of genes carried relative to the total number of genes listed as the response variable and the phylogroup as the explanatory factor. We computed the estimated average number of genes carried by each phylogroup by multiplying the proportion estimated with the model times the total number of genes listed. This estimate was simply equal to the average number of genes observed among the strains of the phylogroup, but using a binomial regression also provided a standard error on these estimates.

As for residence and colonization (see above), the values and standard errors of pathogenicity and the numbers of ARGs or VAGs carried for each phylogroup defined the distributions of these estimates. We used these distributions to generate confidence intervals by parametric bootstrapping. We sampled the distributions to generate one million set of each response variable (pathogenicity, number of ARGs, total number of VAGs, and number of VAGs associated with each type of factor) for each phylogroup. We computed a Spearman’s correlation coefficients between each of these variables and the sets of residence times generated earlier (see above). We considered a correlation to be significant if 95% of the distribution of Spearman’s coefficients was different from zero. Unlike the comparison between residence time and colonization rates explained above, the pairs of characteristics considered (residence time versus pathogenicity or virulence) come from datasets including different strains. Although both datasets involve commensal strains of *E. coli* (when considering the number of ARGs or VAGs) sampled in high-income countries (the same country in the case of the French dataset), these comparisons therefore only provide information at the phylogroup level.

### Description of the model of *E. coli* dynamics

We developed a continuous time, discrete-state Markov chain model describing the dynamics of *E. coli* clones in a host population, integrating findings on colonization rates and residence times (Fig. 1). In this model, each host can contain between 0 and *N* clones, each belonging to one of *G* possible phylogroups. The discrete states of the model are therefore host compositions in *E. coli* clones, which are expressed as unordered multisets of the phylogroups of the clones contained. Hosts can be in one of $M=\left(\genfrac{}{}{0pt}{}{G+N}{G}\right)$ states, which are numbered as follows:


$$ {\displaystyle \begin{array}{l}\begin{array}{l}{S}_1=\varnothing \\{}{S}_2=\left\{1\right\}\\{}{S}_3=\left\{2\right\}\end{array}\\{}\begin{array}{l}\vdots \\{}{S}_{G+1}=\left\{1,1\right\}\\{}{S}_{G+2}=\left\{1,2\right\}\end{array}\\{}\begin{array}{l}\vdots \\{}{S}_M=\overset{\times N}{\overbrace{\left\{G,\cdots, G\right\}}}\end{array}\end{array}}. $$

Hence, state *S*_1_ corresponds to a host with no clone, *S*_2_ to a host with a clone of phylogroup 1, *S*_*G* + 2_ to a host with two clones of phylogroup 1, *S_M_* to a host with *N* clones of phylogroup *G*. The variables of the model are the set of probabilities *P_i_*(*t*) that a host is in state *S_i_* at time *t*.

The transitions between states are defined by the matrix **T**_**t**_ of size *M* × *M*, whose elements, noted *τ_i,j_*(*t*) > 0 for the transition between state *i* and state *j*, are driven by the clearance and colonization of clones, respectively happening at rates ${\mu}_{k,n,{n}_k}$ and *λ_k,n_*(*t*) for clones of phylogroup *k*:


(1) \begin{align*} {\mu}_{k,n,{n}_k}&=1/{e}^{a_k+b\left(n-1\right)+{b}_s\left({n}_k-1\right)} \nonumber \\{}{\lambda}_{k,n}(t)&={F}_k(t)/{e}^{c_k+ dn} \end{align*}

These functional forms are informed by results from the analysis of the epidemiological data presented above (see Results). Clearance is affected by three parameters: *a_k_* is a phylogroup effect, *b* represents the impact of the number of other clones in the host (*n* - 1), and *b_s_* represents the impact of the number of other clones of the same phylogroup (*n_k_* - 1). Negative values of *b* and *b_s_* respectively correspond to faster clearance as more clones are present in the host, and to faster clearance as more clones of the same phylogroup are present. Colonization is affected by the frequency of phylogroup *k* in the host population *F_k_*(*t*) and two parameters: *c_k_* is a phylogroup effect and *d* represents the impact of the number of other clones in the host (*n* - 1). Negative values of *d* correspond to faster colonization as more clones are present. The frequency of the phylogroup *F_k_*(*t*) is defined as:


(2) \begin{equation*} {F}_k(t)=\sum_{i=1}^M{V}_{i,k}{P}_i(t),{V}_{i,k}=\left\{\begin{array}{@{}ll}0& if\ {l}_{i}=1\\{}{m}_{i,k}/{l}_i& if\ {l}_{i}>1\end{array}\right. \end{equation*}

with *m_i,k_* the number of clones of phylogroup *k* in state *i* and *l_i_* the total number of clones in state *i*. Hence, *V_i,k_* corresponds to the proportion of clones of phylogroup *k* shed by a host in state *i*.

The transition matrix **T**_**t**_ is sparse, as the transition rates *τ_i,j_*(*t*) are positive only if a single colonization or clearance event is needed to reach state *j* from state *i*. The rows of **T**_**t**_ sum to 0, as the diagonal element ${\mathrm{\tau}}_{i,i}(t)=-{\sum}_{j\ne i}{\mathrm{\tau}}_{i,j}(t)$. We computed the matrix exponential ${\mathbf{A}}_{\mathbf{t}}={e}^{{\mathbf{T}}_{\mathbf{t}}}$ using the package *expm* [58] to get the transition probabilities between states accounting for potential multiple transitions over a single time-step.

We used this model to investigate the possible coexistence of several bacterial phylogroups in a host population and the impact of public health measures altering the colonization and clearance rates on the hierarchy of phylogroups. We quantified coexistence with two measures. Within-host coexistence *E^W^* ∈ [0, 1] corresponds to the proportions of hosts carrying clones of at least two phylogroups. Inter-host coexistence E^I*^ ∈ [0, ln(*G*)] is a Shannon index on the relative frequencies of the phylogroup in the host population:


(3) \begin{equation*} {E}^I(t)=-\sum_{k=1}^G{f}_k^h(t)\mathit{\ln}\left({f}_k^h(t)\right),{f}_k^h(t)=\frac{F_k^h(t)}{\sum_{k=1}^G{F}_k^h(t)} \end{equation*}

We simulated variations in state probabilities in discrete time with a time-step of a day, by updating the elements of the transition matrix **Tt** using the phylogroup frequencies of the previous day *F_k_*(*t* - 1). In contrast to clearance rates, colonization rates were not constant over the course of the simulation because they depended on the frequency of each phylogroup in the population. We stopped the simulation when a steady state was reached, i.e. for t^*^ such that ${\sum}_{i=1}^N\left|{P}_i\left({t}^{\ast}\right)-{P}_i\left({t}^{\ast }-1\right)\right|<{10}^{-12}$. We considered this steady state as the asymptotic state of our system, with the asymptotic probability of state *S_i_* noted P_i_^*^. We could then also compute the asymptotic within-host coexistence E^W*^ and inter-host coexistence E^I*^ at this steady state.

We considered a simple case of two phylogroups *i* and *j*, characterized by an average colonization-to-clearance ratio $\overline{R_{i,j}}=\left({R}_i+{R}_j\right)/2$ and a ratio difference ${D}_{i,j}={R}_i\hbox{--} {R}_j$. We simulated our model with a time-step of one day until a stationary state was reached (precisely, at time t^*^ such that ${\sum}_{i=1}^N\left|{P}_i\left({t}^{\ast}\right)-{P}_i\left({t}^{\ast }-1\right)\right|<{10}^{-12}$).

## Results

### Determinants of residence time

For both adult datasets, AFT models showed differences in clone residence times according to host and phylogroup (see Table S2 for the French dataset and Table S4 for the USA dataset). They also provided support for greater niche overlap between clones of the same phylogroup than between those of different phylogroups. Indeed, the residence time of a clone was significantly reduced by the presence of other clones in the host, and even more so if these clones were of the same phylogroup (Table 2). According to the analysis of the French dataset, the residence time was divided by 1.45 (95%CI = [1.219,1.717]) for each additional clone of a different phylogroup, and by 2.09 (95%CI = [1.594,2.751]) for each additional clone of the same phylogroup. According to the analysis of the USA dataset, the residence time was divided by 1.68 (95%CI = [1.266,2.358]) for each additional clone of different phylogroup, and by 5.09 (95%CI = [1.800,14.423]) for each additional clone of the same phylogroup. The stronger competition between strains of the same vs. distinct phylogroups suggests niche differentiation between phylogroups in the human gut. The analysis of the USA dataset also showed that the cell density of a clone increased its residence time (Table 2), which was multiplied by 1.32 (95%CI = [1.002,1.499]) when the clone density was multiplied by 10. Neither of the two AFT models selected based on AIC included total cell density as an explanatory factor.

**Table 2:** *Z* scores and *P* values of the coefficients corresponding to the continuous explanatory variables in the AFT models of residence and colonization selected for the analyses of the French and USA datasets. Positive value of *Z* score indicate longer residence times/lower colonization rates as the factor increases. See Supplementary material for the tables including qualitative factors, i.e. host and phylogroup.

Dataset	Response variable	Explanatory factor	*Z* score	*P* value
French	Residence time	Total number of clones	−4.218	< 0.001
USA	Residence time	Total number of clones	−3.444	< 0.001
French	Residence time	Number of clones of the same phylogroup	−2.654	0.008
USA	Residence time	Number of clones of the same phylogroup	−2.135	0.033
USA	Residence time	Log_10_ of cell density	1.983	0.047
French	Colonization rate	Total number of clones	−9.493	< 0.001
USA	Colonization rate	Total number of clones	−9.775	< 0.001

To confirm the robustness of these results to the statistical method used, we performed additional analyses using semi-parametric Cox models with the same explanatory factors as those used for the AFT models, which yielded consistent results (see Supplementary material).

### Determinants of colonization rate

AFT models showed differences in colonization rates according to host and phylogroup for both datasets (see Table S7 for the French dataset and Table S9 for the USA dataset). The two models also showed that the colonization rate was increased by the presence of other clones in the host (Table 2). The colonization rate was predicted to be multiplied by 1.76 (95%CI = [1.562, 1.970]) for each additional clone already present in the host according to the analysis of the French dataset, and by 2.89 (95%CI = [2.342, 3.589]) for each additional clone already present in the host according to the analysis of the USA dataset. However, the impact of other clones already residing in the host on the colonization rate was phylogroup-independent. Indeed, neither of the two AFT models selected based on AIC included the number of clones of the same phylogroup. They also did not select cell density in the host as an explanatory factor.

To confirm the robustness of the results to the statistical method used, we performed additional analyses using semi-parametric Cox models with the same explanatory factors as those used for the AFT models, confirming the results of the AFT models (see Supplementary material).

### Trade-off between colonization and residence

**Figure 2:**
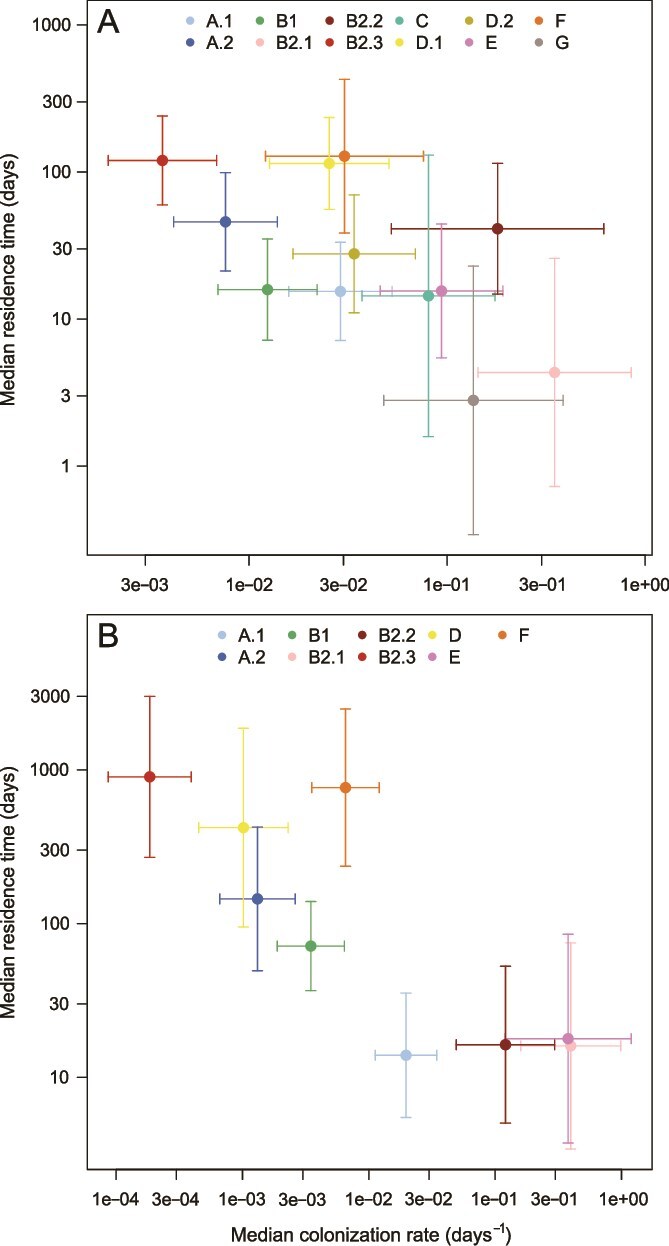
Median residence time as a function of the median colonization rate for each phylogroup appearing in the French (A) and the USA (B) datasets, for the average host and average values of the quantitative explanatory factors in their respective datasets. Estimates (dots) are represented with their 95% confidence interval (bars).

To investigate the relationship between residence and colonization at the phylogroup level, we computed the estimates and standard errors of colonization rate and residence time predicted by the AFT models for each phylogroup (Fig. 2) and quantified the association between them. The two life-history traits were negatively associated across phylogroups, both in France (Spearman’s ρ = −0.615, parametric bootstrapping 95%CI = [−0.769, −0.238], *P* value = 0.002) and in the USA (Spearman’s ρ = −0.783, parametric bootstrapping 95%CI = [−0.917, −0.599], *P* value <0.001). This suggested a negative trade-off between the colonization and residence abilities of phylogroups. In other words, phylogroups were distributed along an axis ranging from those with a fast turnover (strong colonizers with short residence times) to those with a slow turnover (poor colonizers with long residence times). The arrangement of phylogroups along this trade-off was broadly conserved in France and the USA, with slow-turnover phylogroups including B2.3, D, and F; intermediate phylogroups including A.2 and B1, and fast-turnover phylogroups including B2.1 (and B2.2 in the USA only, G in France only).

To further assess the robustness of the negative trade-off, we also analysed colonization using colonization rates corrected by the frequencies of phylogroup in the datasets themselves rather than external data (see Supplementary material). Using estimates from these models, we found similar negative correlations with the residence time, both in the French dataset (Spearman’s ρ = −0.769, parametric bootstrapping 95%CI = [−0.832, −0.336], *P* value <0.001) and in the USA dataset (Spearman’s ρ = −0.733, parametric bootstrapping 95%CI = [−0.900, −0.583], *P* value <0.001).

We investigated whether the trade-off we identified in adults was also observed during the initial gut colonization of infants (see Supplementary material). We analysed an additional longitudinal dataset including infants sampled over a year after birth, analysed in a previous study [36]. The trade-off was also evident in this specific context.

### Correlation between residence, pathogenicity, and VAG

**Figure 3:**
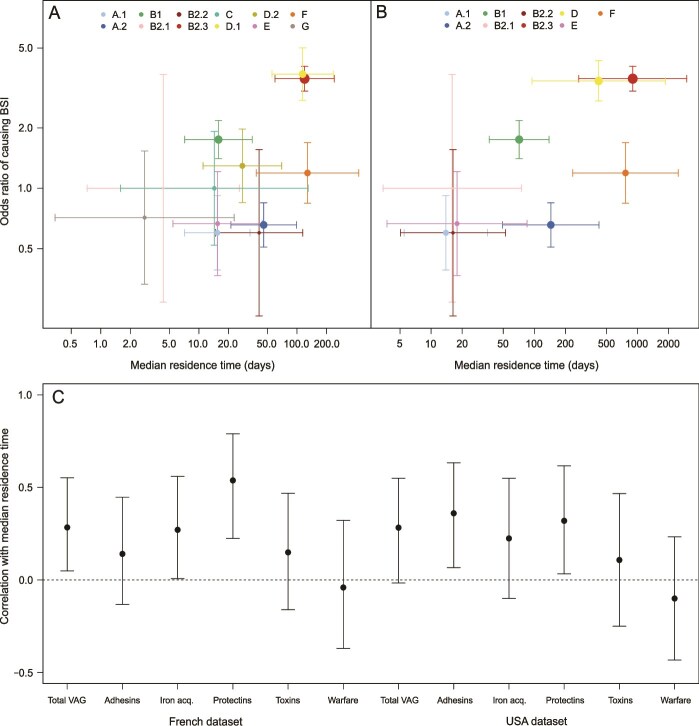
(A and B) Odds ratio of the association with bloodstream infections (BSI) as a function of median residence time for each phylogroup appearing in the French (A) and the USA (B) datasets. Estimates (dots) are represented with their 95% confidence interval (bars), with the size of the dots proportional to the number of samples of a given phylogroup used to estimate the odds ratios of causing BSI. (C) Correlation rate between the median residence time of each phylogroup estimated for the two datasets and its number of VAGs (total or associated with adhesins, iron acquisition systems, protectins, toxins, or bacterial warfare). Estimates (dots) are represented with their parametric bootstrap 95% confidence interval (bars). Correlations whose confidence interval does not include 0 are considered significant.

The trade-off between colonization and residence has implications for the evolution of bacterial virulence, as the residents are also the most pathogenic bacteria and tend to carry more VAGs [55, 56]. The estimated pathogenicity of phylogroups, based on our additional dataset, was positively correlated with residence time for both datasets (French dataset: Spearman’s ρ = 0.518, parametric bootstrapping 95%CI = [0.105, 0.741], *P* value =0.013; USA dataset: Spearman’s ρ =0.745, parametric bootstrapping 95%CI = [0.217, 0.833], *P* value = 0.004) (Fig. 3A, 3B). The total number of VAGs carried was also variable between phylogroups (Kruskal-Wallis χ^2^_df = 11_ = 184.29, *P* value <0.001) and was positively correlated with residence for the French data (Spearman’s ρ = 0.273, parametric bootstrapping 95%CI = [0.049,0.531], *P* value = 0.022) (Fig. 3C). The USA data suggested the same trend (Spearman’s ρ = 0.383, parametric bootstrapping 95%CI = [−0.017, 0.533], *P* value = 0.053). Differences were observed in the strength of the correlation when considering genes associated with specific functions: those linked with protectins were the most strongly correlated with residence, but those linked with toxins were not, overall or only considering those linked to bacterial warfare. Genes involved in iron acquisition (*P* value = 0.047) and adhesins (*P* value = 0.018) were significantly correlated with residence time, for the French and USA datasets respectively. However, we did not find any evidence of correlation between residence time and the number of antibiotic-resistance genes (ARG) carried (Fig. S9), in either the French (*P* value = 0.350) or the USA dataset (*P* value = 0.865).

### Model of bacterial epidemiological dynamics

Following data analysis, we derived the implications of our results for the epidemiological and evolutionary dynamics of *E. coli*. We developed a model describing the dynamics of bacterial clones in a host population, informed by the epidemiological data. In this model, the clearance rate ${\mu}_{k,n,{n}_k}$ of a clone of phylogroup *k* (see Eq. 1) depend on a phylogroup factor *a_k_*, the number of other clones in the host (*n -* 1) with a factor *b* and the number of other clones of the same phylogroup *k* in the host (*n_k_* - 1) by a factor *b_s_*. The colonization rate *λ_k,n_*(*t*) (see Eq. 1) depends on a phylogroup factor *c_k_*, the number of clones in the host (*n*) with a factor *d* and the frequency of the phylogroup in the host population *F_k_*(*t*).

Based on Eq. 1, each phylogroup is characterized by an intrinsic colonization-to-clearance ratio ${R}_k={e}^{a_k-{c}_k}$, which is also the basic reproduction number. Indeed, when a single clone of phylogroup *k* is introduced in a large population of *H* empty hosts, the frequency of this clone is ${F}_k(t)=1/H$, the number of hosts to colonize is approximately *H* and the expected residence time of the clone is $1/{\mu}_{k,1,1}$. Thus, the expected number of hosts colonized after this single introduction is:


(4) \begin{equation*} H{\lambda}_{k,0}(t)\times \frac{1}{\mu_{k,1,1}}=H\frac{\left(1/H\right){e}^{-\left({c}_k+0\times d\right)}}{e^{-\left({a}_k+0\times{b}_s+0\times{b}_o\right)}}={e}^{a_k-{c}_k}={R}_{k.} \end{equation*}

In spite of the complexity of the model, which enables arbitrary multiplicity of colonization and thus a very large number of possible colonization states, simple mathematical results can be derived in some conditions (see Supplementary material). When there is no niche differentiation, that is the intensity of competition within the host is the same irrespective of phylogroup identity of the clones (*b_s =_* 0), the colonization-to-clearance ratio predicts when a phylogroup can invade a resident population at equilibrium. In other words, commensal bacteria evolve to maximize their colonization-to-clearance ratio *R_k_* and the differences in this ratio define a “hierarchy” between phylogroups. The negative trade-off we found in data implied that several phylogroups had similar colonization-to-clearance ratios *R_k_* but different strategies, from fast turnover (high values of *a_k_* and *c_k_*) to slow turnover (low values of *a_k_* and *c_k_*).

**Figure 4:**
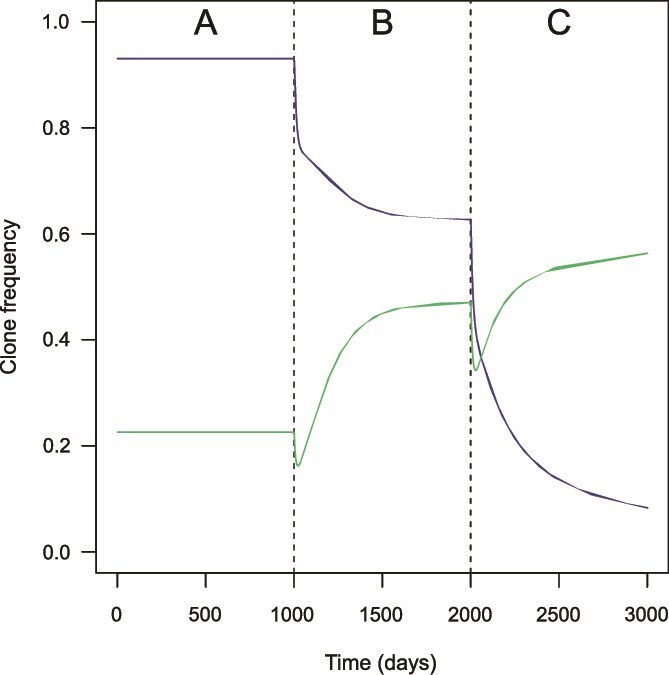
Variation in the frequencies of phylogroups *x* (purple) and *y* (green) over 3000 simulated days of the model with increasing levels of additive clearance (*θ*), with *R_x_* = 3.5, *R_y_* = 3, *b* = 0, *b_s_* = −0.1, and d = 0. Three time-periods of 1000 days each (separated by dashed lines) are defined: no additive clearance (*θ =* 0) for *t* ∈ [0, 1000[(A), *θ =* 0.1 for *t* ∈ [1000, 2000[(B), and *θ =* 0.2 for *t* ∈ [2000, 3000] (C). Phylogroup *x* has a slower turnover than *y*. The phylogroup hierarchy is reversed for a high enough value of *θ*.

We explored with our mathematical model how the hierarchy between phylogroups is affected by non-phylogroup-specific environmental change or public hygiene measure (see Supplementary material). The hierarchy is not affected by a “multiplicative” change, multiplying the colonization or clearance rate by a constant factor. In contrast, a new, independent source of clearance can alter the hierarchy of phylogroups by disproportionately affecting the phylogroups with a slower turnover. These changes in hierarchy can translate into variations in phylogroup frequencies. For instance, a progressive increase in external rate of clearance eventually leads to the dominance of a fast-turnover over a slow-turnover phylogroup (Fig. 4).

### Conditions for coexistence of multiple phylogroups

We assessed the conditions in which our model enables coexistence between two phylogroups, depending on their respective colonization-to-clearance ratios. We considered a simple case of two phylogroups *i* and *j*, characterized by an average colonization-to-clearance ratio $\overline{R_{i,j}}=\left({R}_i+{R}_j\right)/2$ and a ratio difference ${D}_{i,j}={R}_i-{R}_j$. We simulated our model with a time-step of one day until a stationary state was reached, and computed the asymptotic within-host coexistence E^W*^ and inter-host coexistence E^I*^ at the steady state.

**Figure 5:**
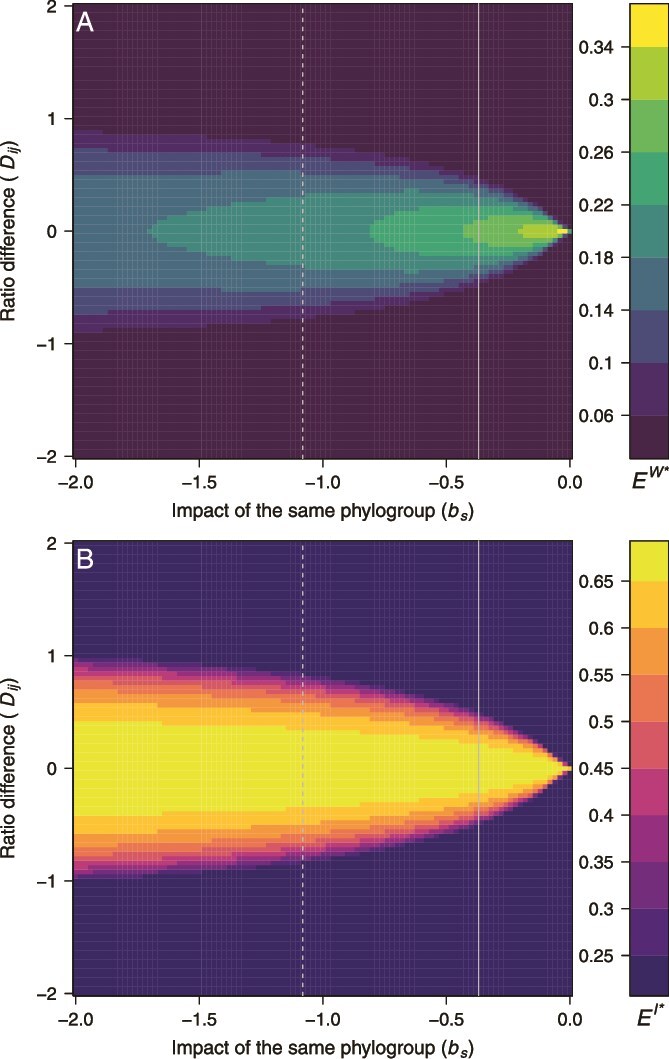
Asymptotic within-host coexistence E^W*^ (A) and inter-host coexistence E^I*^ (B) as a function of the ratio difference *D_ij_* and the strength of clones of the same phylogroup on clearance *b_s_*. Vertical lines correspond to the coefficients characterizing the impact of other clones of the same phylogroup from the best AFT models for the French (solid) and USA datasets (dashed). For these simulations, $\overline{R_{ij}}$ = 2, *b* = 0, *d* = 0, E^W*^ ∈ ]0, 1] and E^I*^ ∈ ]0, ln(2)].

These simulations revealed that only structurally unstable coexistence is possible if there is no niche differentiation (*b_s_* = 0, see Eq. 1). This is in line with the mathematical analysis of the model (Supplementary material). In that case, coexistence is maintained only if the two phylogroups have exactly the same colonization-to-clearance ratio (*D_i,j_* = 0). Stable coexistence is possible when there is additional competition between clones of the same phylogroup (*b_s_* < 0). As competition within phylogroups increases (more negative *b_s_*), the range of values of *D_i,j_* for which coexistence is stable widens, and within-host coexistence also declines (Fig. 5A). Indeed, more negative values of *b_s_* lead to higher average clearance rates overall, thus decreasing overall the number of clones per host and the value of E^W*^. Inter-host coexistence was higher as *b_s_* was more negative (Fig. 5B).

The simulation results presented here correspond to $\overline{R_{ij}}$ = 2, *b* = 0, and *d* = 0, and similar results were obtained for other parameter values. Higher values of $\overline{R_{ij}}$ (the average colonization-to-extinction ratio), more positive values of *b* (lower clearance rate as the number of clones in the host increases), and more negative values of *d* (higher colonization rate as the number of clones in the host increases) all resulted in a higher overall number of clones per host, and therefore wider ranges of *D_i,j_* for which coexistence was stable for a given value of *b_s_*. Lower values of $\overline{R_{i,j}}$, more negative values of *b* or more positive values of *d* had the opposite effect.

## Discussion

By analysing longitudinal datasets on *E. coli* gut colonization dynamics in healthy adults from high-income countries, we identified two mechanisms explaining the stable coexistence of *E. coli* phylogroups. The first one is the trade-off between the colonization and residence abilities at the phylogroup level. Our results indicate that the “resident” or “transient” nature of *E. coli* clones, which has been observed for over 100 years [39, 59], is a property partly explained by phylogroups and inversely correlated with the ability to colonize hosts. This trade-off is equalizing *sensu* Chesson [60], meaning that it reduces differences in fitness between the phylogroups. In our model of colonization dynamics with human-to-human transmission, fitness is mathematically equal to the colonization-to-clearance ratio *R_k_*. Therefore, the coexistence of multiple phylogroups in a population at equilibrium implies that these phylogroups compensate shorter residence times with a higher colonization rate (or vice versa) to achieve similar fitness. Indeed, clones with lower fitness would not rise in frequency, whereas clones with larger fitness would invade and replace all other clones. This trade-off was also evidenced in infants followed during their first year of life, who have much simpler microbiome [61], suggesting that it may not depend on the presence of other species.

The second mechanism explaining the stable coexistence of *E. coli* phylogroups is niche differentiation, which acts as a stabilizing mechanism that can explain long-term coexistence [60]. In both datasets, residence is shorter when a clone is in a host together with another clone of the same phylogroup. This indicates stronger competition within phylogroups than between phylogroups, which could be explained by a greater overlap of ecological niches between clones that are phylogenetically closer. Colonization does not exhibit the same dependence to the number of clones of the same phylogroup. This suggests that niche overlap only affects clones after the colonization of a new host.

Our analyses also highlighted substantial heterogeneity between hosts in bacterial dynamics, consistent with the absence of “core resident microbiome” reported previously [44] using the USA data. Clone turnover rates varied not only between hosts, but also over time within the same host. Both clearance and colonization were more frequent in hosts with a greater number of different clones. This was particularly apparent in the USA dataset, in which hosts experienced periods of stability with few clones, alternating with rapid successions of colonization and clearance events, during which the number of clones present temporarily increased. These periods of instability could be triggered by temporal variations in the suitability of the host for colonization or residence. They could also be triggered by the temporary destabilization of the microbial community due to the colonization of several clones in a short time span. These explanations are not exclusive and could both contribute to temporal variability. These transient periods of instability have also been observed over decades in a single host, with long periods of residence of clones, mainly of phylogroup B2, interrupted by rapid successions of clones, mainly of phylogroup B1 or A [42].

Although this study focuses on commensal *E. coli*, our results also have implications for the evolution of pathogenicity. Phylogroups have been shown to exhibit consistent differences in their virulence profile and virulence-associated gene content [62, 63], resistance, and pathogenicity [47, 64]. Our results highlight large phylogroup-level differences in commensal traits linked to pathogenicity, confirming the relevance of this level of granularity in *E. coli*. Previous studies have highlighted phylogroup B2 in particular, mainly represented in our data by subgroup B2.3 (Fig. S1), as both resident and virulent [29, 65, 66]. We present a more general positive correlation between the residence time of a phylogroup, and both its pathogenicity and its number of VAG. These results are consistent with the hypothesis that the virulence of *E. coli* as an opportunistic pathogen results from traits linked with its residence ability as a commensal [29, 67]. Our additional analyses focusing on genes associated with specific functions show that protectins, and to a lesser extent adhesins and iron acquisition systems, are positively correlated with residence, whereas the signal was unclear for toxins. Although the most resident phylogroups were identified as the most likely to generate extraintestinal infections, better colonizing phylogroups such as B1, A.1, or E have been associated with pathovars causing severe and acute diarrhoea [43]. These pathovars may benefit more from the ability to colonize rather than from the ability to reside.

Our results might also have implications for the evolution of *E. coli* in response to changes in the environment or public health measures. Multiplicative changes increasing overall clearance rate (or reducing overall transmission) rate by a given factor are not predicted to alter the fitness hierarchy between phylogroups. In contrast, “additive” changes creating new sources of clearance, such as antibiotics, should favour the best colonizers at the expense of the residents. This could explain the resilience of the microbiota in settings with high antibiotic use [68]. Conversely, a more stable gut habitat could explain an increase in the frequency of phylogroup B2, as observed in France [25, 69]. The disproportionate impact of antibiotics on the resident phylogroups could favour the colonisers, which tend to be less extraintestinal pathogenic. It could also generate stronger selection for antibiotic resistance among residents [70]. Yet, we found no link between residence and the presence of resistance genes in our data [25, 69]. This differs, e.g. from *S. pneumoniae*, another human commensal bacteria and opportunistic pathogen, where serotypes carried for longer are also more antibioresistant [70].

Our study has limitations. We focused on colonization and clearance rates, which likely result from many underlying mechanisms, such as resource uptake, bacterial warfare, or predation by phages [71]. Moreover, residence as measured in our study corresponds to the ability of a clone to maintain at densities high enough to be detected in the dominant flora. The dynamics of subdominant clones remains unobserved and would be interesting to quantify in further studies. Increasing the number of clones characterized by sample is expected to improve the completeness of the diversity observed in the data, but at a cost, for instance on the number of hosts included. Our results are supported by congruent analyses on two complementary datasets, focusing respectively on a large number of hosts (French) or on a large number of clones characterized per sample (USA). To test whether sampling a limited number of clones could bias our results or limit power, we randomly sub-sampled the USA dataset to five clones per sample (see Supplementary material). Neither the trade-off nor the niche differentiation was biased by sub-sampling. The trade-off was still robustly identified, with a consistent negative correlation between residence and colonization similar to the one observed with the complete data, whereas the statistical power to detect niche differentiation was reduced, presumably because of the reduced diversity in sub-sampled data.

Colonization encompasses all events between the shedding of a clone by a host and its detection at high enough densities in another host. In our model, this series of events is encapsulated in the colonization rate. This is a valid description if the time spent by bacterial cells in the environment between hosts is short. In this model, better survival in the environment would result in a higher colonization rate. However, our model cannot account for long residence time in the environment or non-human hosts, which are known to harbour distinct *E. coli* clones [72, 73] with dynamics potentially largely independent from the human hosts. We investigated how transmission of clones from an external environmental reservoir would affect the outcome of our model (see Supplementary material). Results show that an external reservoir could stabilize coexistence by providing a potential additional source of rare strains in the population.

We chose to characterize the commensal traits of *E. coli* at the phylogroup level, which is a coarser-grained level than studies focusing, for instance, on sequence type complexes (STc) [e.g. 20, 69] or genome-wide association studies [54, 74], that aim at linking specific genes and bacterial phenotypes. This approach is consistent with our objective of characterizing and explaining the maintenance of the diversity of *E. coli* at the species scale, and the coarse-grained level of the traits we consider (residence and colonization), which result themselves from multiple interacting mechanisms. However, we could not examine the existing within-phylogroup variability with this data, nor investigate the link between the differences in turnover speed observed and specific genes or functions. Our analysis of additional data about pathogenicity and virulence gene carriage unveiled correlations between VAGs and residence abilities as a commensal hypothesized in previous studies [28], but strictly at the phylogroup level. Complementary genome-based analyses of our dataset will allow to confirm this result, to study the mechanisms underlying the trade-off and the mechanisms underlying niche differentiation.

In conclusion, by analysing longitudinal data on *E. coli* epidemiological dynamics in healthy adults from two separate datasets, we discovered general principles structuring the diversity of this species: major lineages align along a colonization-residence trade-off equalizing their fitness, and niche differentiation stabilizes their long-term coexistence. We also discovered a strong correlation between residence and pathogenicity. Our results on high-income countries, where the phylogroup with the slowest turnover B2 is over-represented [43, 75], would be nicely complemented by longitudinal analyses in other countries with radically different phylogroup frequencies. Finally, our work also suggests mechanisms by which the micro-evolutionary dynamics of *E. coli* can be altered, and opens perspective for the study of epidemiological and evolutionary dynamics of other human commensal bacteria and opportunistic pathogens.

## Supplementary Material

supplementary_wraf089

## Data Availability

The datasets uses for the analyses presented in the study are available in the following Zenodo repository: doi:10.5281/zenodo.14018576.

## References

[1] Fraser C, Hollingsworth TD, Chapman R. et al. Variation in HIV-1 set-point viral load: epidemiological analysis and an evolutionary hypothesis. *Proc Natl Acad Sci* 2007;104:17441–6. 10.1073/pnas.0708559104 17954909 PMC2077275

[2] Fraser C, Alm EJ, Polz MF. et al. The bacterial species challenge: making sense of genetic and ecological diversity. *Science* 2009;323:741–6. 10.1126/science.1159388 19197054

[3] Turner PE, Souza V, Lenski RE. Tests of ecological mechanisms promoting the stable coexistence of two bacterial genotypes. *Ecology* 1996;77:2119–29. 10.2307/2265706

[4] Ferenci T . Trade-off mechanisms shaping the diversity of bacteria. *Trends Microbiol* 2016;24:209–23. 10.1016/j.tim.2015.11.009 26705697

[5] Baquero F, Coque TM, Galán JC. et al. The origin of niches and species in the bacterial world. *Front Microbiol* 2021;12:657986. 10.3389/fmicb.2021.657986 33815348 PMC8010147

[6] Sieben AJ, Mihaljevic JR, Shoemaker LG. Quantifying mechanisms of coexistence in disease ecology. *Ecology* 2022;103:e3819. 10.1002/ecy.3819 35855596

[7] Cobey S, Lipsitch M. Niche and neutral effects of acquired immunity permit coexistence of pneumococcal serotypes. *Science* 2012;335:1376–80. 10.1126/science.1215947 22383809 PMC3341938

[8] Hanage WP, Spratt BG, Turner KM. et al. Modelling bacterial speciation. *Philos Trans R Soc B Biol Sci* 2006;361:2039–44. 10.1098/rstb.2006.1926 PMC1764933 17062418

[9] Tenaillon O, Skurnik D, Picard B. et al. The population genetics of commensal *Escherichia coli*. *Nat Rev Microbiol* 2010;8:207–17. 10.1038/nrmicro2298 20157339

[10] Kotloff KL, Nataro JP, Blackwelder WC. et al. Burden and aetiology of diarrhoeal disease in infants and young children in developing countries (the global enteric multicenter study, GEMS): a prospective, case-control study. *Lancet* 2013;382:209–22. 10.1016/S0140-6736(13)60844-2 23680352

[11] Kotloff KL, Platts-Mills JA, Nasrin D. et al. Global burden of diarrheal diseases among children in developing countries: incidence, etiology, and insights from new molecular diagnostic techniques. *Vaccine* 2017;35:6783–9. 10.1016/j.vaccine.2017.07.036 28765005

[12] Zhang L, Foxman B. Molecular epidemiology of *Escherichia coli* mediated urinary tract infections. *Front Biosci-Landmark* 2003;8:e235–44. 10.2741/1007 12456300

[13] Foxman B . The epidemiology of urinary tract infection. *Nat Rev Urol* 2010;7:653–60. 10.1038/nrurol.2010.190 21139641

[14] De Kraker ME, Davey PG, Grundmann H. et al. Mortality and hospital stay associated with resistant *Staphylococcus aureus* and *Escherichia coli* bacteremia: estimating the burden of antibiotic resistance in Europe. *PLoS Med* 2011;8:e1001104. 10.1371/journal.pmed.1001104 22022233 PMC3191157

[15] Goto M, McDanel JS, Jones MM. et al. Antimicrobial nonsusceptibility of gram-negative bloodstream isolates, veterans health administration system, United States, 2003–2013. *Emerg Infect Dis* 2017;23:1815–25. 10.3201/eid2311.161214 29047423 PMC5652419

[16] Liu L, Johnson HL, Cousens S. et al. Global, regional, and national causes of child mortality: an updated systematic analysis for 2010 with time trends since 2000. *Lancet* 2012;379:2151–61. 10.1016/S0140-6736(12)60560-1 22579125

[17] Abernethy JK, Johnson AP, Guy R. et al. Thirty day all-cause mortality in patients with *Escherichia coli* bacteraemia in England. *Clin Microbiol Infect* 2015;21:251.e1–8. 10.1016/j.cmi.2015.01.001 25698659

[18] Feldman SF, Temkin E, Wullfhart L. et al. A nationwide population-based study of *Escherichia coli* bloodstream infections: incidence, antimicrobial resistance and mortality. *Clin Microbiol Infect* 2022;28:879.e1–7. 10.1016/j.cmi.2021.12.009 34922002

[19] Clermont O, Condamine B, Dion S. et al. The E phylogroup of *Escherichia coli* is highly diverse and mimics the whole *E. coli* species population structure. *Environ Microbiol* 2021;23:7139–51. 10.1111/1462-2920.15742 34431197

[20] Chauhan SM, Ardalani O, Hyun JC. et al. Decomposition of the pangenome matrix reveals a structure in gene distribution in the *Escherichia coli* species. *mSphere* 2025;10:e00532–24. 10.1128/msphere.00532-24 39745367 PMC11774025

[21] Clermont O, Bonacorsi S, Bingen E. Rapid and simple determination of the *Escherichia coli* phylogenetic group. *Appl Environ Microbiol* 2000;66:4555–8. 10.1128/AEM.66.10.4555-4558.2000 11010916 PMC92342

[22] Clermont O, Christenson JK, Denamur E. et al. The Clermont *Escherichia coli* phylo-typing method revisited: improvement of specificity and detection of new phylo-groups. *Environ Microbiol Rep* 2013;5:58–65. 10.1111/1758-2229.12019 23757131

[23] Meier-Kolthoff JP, Hahnke RL, Petersen J. et al. Complete genome sequence of DSM 30083T, the type strain (U5/41T) of *Escherichia coli*, and a proposal for delineating subspecies in microbial taxonomy. *Stand Genomic Sci* 2014;9:2. 10.1186/1944-3277-9-2 25780495 PMC4334874

[24] Carlos C, Pires MM, Stoppe NC. et al. *Escherichia coli* phylogenetic group determination and its application in the identification of the major animal source of fecal contamination. *BMC Microbiol* 2010;10:161. 10.1186/1471-2180-10-161 20515490 PMC2889953

[25] Massot M, Daubié AS, Clermont O. et al. Phylogenetic, virulence and antibiotic resistance characteristics of commensal strain populations of *Escherichia coli* from community subjects in the Paris area in 2010 and evolution over 30 years. *Microbiology* 2016;162:642–50. 10.1099/mic.0.000242 26822436 PMC6365622

[26] Köhler C-D, Dobrindt U. What defines extraintestinal pathogenic *Escherichia coli*? *Int J Med Microbiol* 2011;301:642–7. 10.1016/j.ijmm.2011.09.006 21982038

[27] Denamur E, Clermont O, Bonacorsi S. et al. The population genetics of pathogenic *Escherichia coli*. *Nat Rev Microbiol* 2021;19:37–54. 10.1038/s41579-020-0416-x 32826992

[28] Wold AE, Caugant DA, Lidin-Janson G. et al. Resident colonic *Escherichia coli* strains frequently display uropathogenic characteristics. *J Infect Dis* 1992;165:46–52. 10.1093/infdis/165.1.46 1727897

[29] Le Gall T, Clermont O, Gouriou S. et al. Extraintestinal virulence is a coincidental by-product of commensalism in B2 phylogenetic group *Escherichia coli* strains. *Mol Biol Evol* 2007;24:2373–84. 10.1093/molbev/msm172 17709333

[30] Vosti KL . A prospective, longitudinal study of the behavior of serologically classified isolates of *Escherichia coli* in women with recurrent urinary tract infections. *J Inf Secur* 2007;55:8–18. 10.1016/j.jinf.2007.01.006 17331583

[31] Fang X, Monk JM, Nurk S. et al. Metagenomics-based, strain-level analysis of *Escherichia coli* from a time-series of microbiome samples from a Crohn’s disease patient. *Front Microbiol* 2018;9:2559. 10.3389/fmicb.2018.02559 30425690 PMC6218438

[32] Seidman JC, Johnson LB, Levens J. et al. Longitudinal comparison of antibiotic resistance in diarrheagenic and non-pathogenic *Escherichia coli* from young Tanzanian children. *Front Microbiol* 2016;7:1420. 10.3389/fmicb.2016.01420 27656179 PMC5013055

[33] Brodrick HJ, Raven KE, Kallonen T. et al. Longitudinal genomic surveillance of multidrug-resistant *Escherichia coli* carriage in a long-term care facility in the United Kingdom. *Genome Med* 2017;9:70. 10.1186/s13073-017-0457-6 28738847 PMC5525225

[34] Adlerberth I, Jalil F, Carlsson B. et al. High turnover rate of *Escherichia coli* strains in the intestinal flora of infants in Pakistan. *Epidemiol Infect* 1998;121:587–98. 10.1017/S0950268898001484 10030708 PMC2809566

[35] Nowrouzian F, Hesselmar B, Saalman R. et al. *Escherichia coli* in infants’ intestinal microflora: colonization rate, strain turnover, and virulence gene carriage. *Pediatr Res* 2003;54:8–14. 10.1203/01.PDR.0000069843.20655.EE 12700366

[36] Östblom A, Adlerberth I, Wold AE. et al. Pathogenicity island markers, virulence determinants malX and usp, and the capacity of *Escherichia coli* to persist in infants’ commensal microbiotas. *Appl Environ Microbiol* 2011;77:2303–8. 10.1128/AEM.02405-10 21317254 PMC3067437

[37] Mäklin T, Thorpe HA, Pöntinen AK. et al. Strong pathogen competition in neonatal gut colonisation. *Nat Commun* 2022;13:7417. 10.1038/s41467-022-35178-5 36456554 PMC9715557

[38] Sears HJ, Brownlee I, Uchiyama JK. Persistence of individual strains of *Escherichia coli* in the intestinal tract of man. *J Bacteriol* 1950;59:293–301. 10.1128/jb.59.2.293-301.1950 15421958 PMC385752

[39] Sears H, Brownlee I. Further observations on the persistence of individual strains of *Escherichia coli* in the intestinal tract of man. *J Bacteriol* 1952;63:47–57. 10.1128/jb.63.1.47-57.1952 14927548 PMC169921

[40] Teunis PF, Evers EG, Hengeveld PD. et al. Time to acquire and lose carriership of ESBL/pAmpC producing *E. coli* in humans in the Netherlands. *PLoS One* 2018;13:e0193834. 10.1371/journal.pone.0193834 29561861 PMC5862452

[41] Calderón D, Cárdenas PA, Prado-Vivar B. et al. A longitudinal study of dominant *E. coli* lineages and antimicrobial resistance in the gut of children living in an upper middle-income country. *J Glob Antimicrob Res* 2022;29:136–40. 10.1016/j.jgar.2022.03.002 PMC9232985 35283334

[42] Condamine B, Morel-Journel T, Tesson F. et al. Strain phylogroup and environmental constraints shape *Escherichia coli* dynamics and diversity over a 20-year human gut time series. *ISME J* 2025;19:wrae245. 10.1093/ismejo/wrae245 39665373 PMC11728103

[43] Escobar-Páramo P, Grenet K, Le Menac'h A. et al. Large-scale population structure of human commensal *Escherichia coli* isolates. *Appl Environ Microbiol* 2004;70:5698–700. 10.1128/AEM.70.9.5698-5700.2004 15345464 PMC520916

[44] Martinson JN, Pinkham NV, Peters GW. et al. Rethinking gut microbiome residency and the Enterobacteriaceae in healthy human adults. *ISME J* 2019;13:2306–18. 10.1038/s41396-019-0435-7 31089259 PMC6776003

[45] Caméléna F, Birgy A, Smail Y. et al. Rapid and simple universal *Escherichia coli* genotyping method based on multiple-locus variable-number tandem-repeat analysis using single-tube multiplex PCR and standard gel electrophoresis. *Appl Environ Microbiol* 2019;85:e02812–8. 10.1128/AEM.02812-18 30610078 PMC6414366

[46] Clermont O, Dixit OV, Vangchhia B. et al. Characterization and rapid identification of phylogroup G in *Escherichia coli*, a lineage with high virulence and antibiotic resistance potential. *Environ Microbiol* 2019;21:3107–17. 10.1111/1462-2920.14713 31188527

[47] Royer G, Darty MM, Clermont O. et al. Phylogroup stability contrasts with high within sequence type complex dynamics of *Escherichia coli* bloodstream infection isolates over a 12-year period. *Genome Med* 2021;13:77. 10.1186/s13073-021-00892-0 33952335 PMC8097792

[48] Mohapatra B, Mazumder A. Comparative efficacy of five different rep-PCR methods to discriminate *Escherichia coli* populations in aquatic environments. *Water Sci Technol* 2008;58:537–47. 10.2166/wst.2008.424 18725719

[49] RCoreTeam . R: A Language and Environment for Statistical Computing, Vol. 2018. Vienna, Austria, 2018.

[50] Therneau TM . A Package for Survival Analysis in R, Vol. 2022. Vienna, Austria, 2022.

[51] Wei L-J . The accelerated failure time model: a useful alternative to the cox regression model in survival analysis. *Stat Med* 1992;11:1871–9. 10.1002/sim.4780111409 1480879

[52] Akaike H . A new look at the statistical model identification. *IEEE Trans Autom Control* 1974;19:716–23. 10.1109/TAC.1974.1100705

[53] Burgaya J, Marin J, Royer G. et al. The bacterial genetic determinants of *Escherichia coli* capacity to cause bloodstream infections in humans. *PLoS Genet* 2023;19:e1010842. 10.1371/journal.pgen.1010842 37531401 PMC10395866

[54] Galardini M, Clermont O, Baron A. et al. Major role of iron uptake systems in the intrinsic extra-intestinal virulence of the genus *Escherichia* revealed by a genome-wide association study. *PLoS Genet* 2020;16:e1009065. 10.1371/journal.pgen.1009065 33112851 PMC7592755

[55] Johnson JR . Virulence factors in *Escherichia coli* urinary tract infection. *Clin Microbiol Rev* 1991;4:80–128. 10.1128/cmr.4.1.80 1672263 PMC358180

[56] Johnson JR, Russo TA. Molecular epidemiology of extraintestinal pathogenic Escherichia coli. *EcoSal Plus* 2018;8:10–1128. 10.1128/ecosalplus.esp-0004-2017 PMC11575673 29667573

[57] Granato ET, Meiller-Legrand TA, Foster KR. The evolution and ecology of bacterial warfare. *Curr Biol* 2019;29:R521–37. 10.1016/j.cub.2019.04.024 31163166

[58] Goulet V, Dutang C, Maechler M. et al. Expm: Matrix Exponential, Log, ‘Etc’, Vol. 2021. Vienna, Austria, 2021.

[59] Totsuka K . Studien über bacterium coli. *Z Für Hyg Infekt* 1903;45:115–24. 10.1007/BF02217018

[60] Chesson P . Mechanisms of maintenance of species diversity. *Annu Rev Ecol Syst* 2000;31:343–66. 10.1146/annurev.ecolsys.31.1.343

[61] Yatsunenko T, Rey FE, Manary MJ. et al. Human gut microbiome viewed across age and geography. *Nature* 2012;486:222–7. 10.1038/nature11053 22699611 PMC3376388

[62] Royer G, Clermont O, Marin J. et al. Epistatic interactions between the high pathogenicity island and other iron uptake systems shape *Escherichia coli* extra-intestinal virulence. *Nat Commun* 2023;14:3667. 10.1038/s41467-023-39428-y 37339949 PMC10282060

[63] Escobar-Páramo P, Clermont O, Blanc-Potard AB. et al. A specific genetic background is required for acquisition and expression of virulence factors in *Escherichia coli*. *Mol Biol Evol* 2004;21:1085–94. 10.1093/molbev/msh118 15014151

[64] Touchon M, Perrin A, De Sousa JAM. et al. Phylogenetic background and habitat drive the genetic diversification of *Escherichia coli*. *PLoS Genet* 2020;16:e1008866. 10.1371/journal.pgen.1008866 32530914 PMC7314097

[65] Nowrouzian FL, Wold AE, Adlerberth I. *Escherichia coli* strains belonging to phylogenetic group B2 have superior capacity to persist in the intestinal microflora of infants. *J Infect Dis* 2005;191:1078–83. 10.1086/427996 15747243

[66] Nowrouzian F, Östblom AE, Wold AE. et al. Phylogenetic group B2 *Escherichia coli* strains from the bowel microbiota of Pakistani infants carry few virulence genes and lack the capacity for long-term persistence. *Clin Microbiol Infect* 2009;15:466–72. 10.1111/j.1469-0691.2009.02706.x 19260873

[67] Johnson JR, Clabots C, Porter SB. et al. Intestinal persistence of colonizing *Escherichia coli* strains, especially ST131-H 30, in relation to bacterial and host factors. *J Infect Dis* 2022;225:2197–207. 10.1093/infdis/jiab638 34979558 PMC9200155

[68] Bich VTN, Le NG, Barnett D. et al. Moderate and transient impact of antibiotic use on the gut microbiota in a rural Vietnamese cohort. *Sci Rep* 2022;12:20189. 10.1038/s41598-022-24488-9 36424459 PMC9691687

[69] Marin J, Clermont O, Royer G. et al. The population genomics of increased virulence and antibiotic resistance in human commensal *Escherichia coli* over 30 years in France. *Appl Environ Microbiol* 2022;88:e0066422–2. 10.1128/aem.00664-22 35862685 PMC9361829

[70] Lehtinen S, Blanquart F, Croucher NJ. et al. Evolution of antibiotic resistance is linked to any genetic mechanism affecting bacterial duration of carriage. *Proc Natl Acad Sci* 2017;114:1075–80. 10.1073/pnas.1617849114 28096340 PMC5293062

[71] Martinson JN, Walk ST. *Escherichia coli* residency in the gut of healthy human adults. *EcoSal Plus* 2020;9:9. 10.1128/ecosalplus.ESP-0003-2020 PMC7523338 32978935

[72] Ludden C, Raven KE, Jamrozy D. et al. One health genomic surveillance of *Escherichia coli* demonstrates distinct lineages and mobile genetic elements in isolates from humans versus livestock. *MBio* 2019;10:e02693–18. 10.1128/mbio.02693-18 30670621 PMC6343043

[73] Money P, Kelly AF, Gould SWJ. et al. Cattle, weather and water: mapping *Escherichia coli* O157: H7 infections in humans in England and Scotland. *Environ Microbiol* 2010;12:2633–44. 10.1111/j.1462-2920.2010.02293.x 20642796

[74] Lees JA, Croucher NJ, Goldblatt D. et al. Genome-wide identification of lineage and locus specific variation associated with pneumococcal carriage duration. *elife* 2017;6:e26255. 10.7554/eLife.26255 28742023 PMC5576492

[75] De SN, Silva JS, Carlos C. et al. Worldwide phylogenetic group patterns of *Escherichia coli* from commensal human and wastewater treatment plant isolates. *Front Microbiol* 2017;8:2512. 10.3389/fmicb.2017.02512 29312213 PMC5742620

